# Hydrothermal synthesis of Zingiber/ZnO for enhanced photodegradation of ofloxacin antibiotic and reactive red azo dye (RR141)

**DOI:** 10.1371/journal.pone.0300402

**Published:** 2024-05-28

**Authors:** David Nugroho, Khemika Wannakan, Suwat Nanan, Rachadaporn Benchawattananon

**Affiliations:** 1 Department of Integrated Science, Faculty of Science, Khon Kaen University, Khon Kaen, Thailand; 2 Materials Chemistry Research Center, Department of Chemistry and Center of Excellence for Innovation in Chemistry (PERCH-CIC), Faculty of Science, Khon Kaen University, Khon Kaen, Thailand; Saveetha Institute of Medical and Technical Sciences: Saveetha University, INDIA

## Abstract

The examination of photocatalyst powders for the total removal of pollutants from aqueous solutions is a vital research subject within the realm of environmental preservation. The objective of this study is to develop a photocatalyst heterojunction consisting of Zingiber/ZnO-H for the degradation of both the reactive red dye (RR 141) and ofloxacin antibiotic in wastewater. The current investigation outlines the process of synthesising a composite material by combining *Zingiber montanum* extract with zinc oxide (ZnO) by a hydrothermal method. The synthesis was conducted at a temperature of 180°C for a period of 4 hours. Consequently. The photocatalyst with a constructed heterojunction shown a notable enhancement in its photocatalytic activity as a result of the improved efficiency in charge separation at the interface. The application of economically viable solar energy facilitated the complete eradication of harmful pollutants through the process of detoxification. The removal of impurities occurs by a process that follows a first-order kinetics. Among the pollutants, RR141 demonstrates the greatest rate constant at 0.02 min-1, while ofloxacin has a rate constant of 0.01 min-1. The assessment of the stability of the produced photocatalyst was conducted after undergoing five cycles. This study additionally investigated the influence of sunshine on degradation, uncovering degradation rates of 97% for RR141 and 99% for ofloxacin when exposed to UV Lamp, and degradation rates of 97% for RR141 and 95% for ofloxacin when exposed to Solar Light.

## 1. Introduction

Current scientific research shows that industrial wastewater contains a significant amount of azo color, specifically the RR141 variant, which has a chemically inherent structure resistant to change [1–5]. The complete identification of hazardous compounds associated with the degradation of permanent dyes remains uncertain, as indicated by previous research findings [6–8]. Therefore, it is crucial to promptly eliminate hazardous pigments from freshwater reservoirs. Fluoroquinolone-based antibiotics have been widely utilized for a prolonged duration to treat bacterial infections. Upon administration, these antibiotics are discharged into the aquatic habitat [9–11]. Recent research has revealed that living organisms exhibit incomplete metabolic processes when it comes to antibiotics [12–15]. Furthermore, it is important to acknowledge that the medications exhibit a lack of biodegradability, resulting in the accumulation of antibiotic residues in water sources. Therefore, it is crucial to ensure a comprehensive degradation of antibiotic contamination with a fluoroquinolone base. Semiconductor photocatalysts have emerged as a subject of significant scientific interest due to their potential for water and air purification, unlike current methods used for pollution treatment [16, 17]. Sample aggregation has been detected in the production of photocatalytic nanostructures when crystal development is inadequately regulated, hindering the implementation of clean technology [18–22]. To effectively address the problem, the physical structure and photocatalytic properties of the catalyst are controlled in the early stages using various covering agents [23–26]. The ZnO photocatalyst has garnered significant interest due to its exceptional transport properties, efficient manufacturing, and versatile form [27–31]. Since ancient times, people have known Zingiber montanum for its content of zingiberene, gingerols, shogaols, and paradols, which act as antioxidants, and have used it in various applications. Zingiber montanum also contains several minerals, such as zinc, iron, and magnesium, which have wide applications as natural mineral ingredients [31–33]. Two main factors limit the practical application of ZnO: inadequate performance under sunlight conditions and vulnerability to photocorrosion. Researchers have employed several techniques to produce ZnO. Precipitation chemistry is a well-known process for producing the semiconducting nanomaterial, ZnO, which offers benefits such as low cost, high efficiency, and good control. In theory, inadequate dissociation of electron-hole pairs in ZnO hampers its photocatalytic efficiency, thereby reducing its ability to absorb ultraviolet rays. (UV). The enhancement of intrinsic characteristics of ZnO has been achieved using two approaches, namely the introduction of dopants or surface modification as well as the fabrication of heterostructures [33–42]. The expected outcome of incorporating noble metals into ZnO is an enhancement in visible light absorption. The observed results can be attributed to the phenomenon of surface plasmon resonance exhibited by noble metals. Furthermore, metal-modified ZnO exhibits a prolonged lifespan of photo-induced charges, leading to enhanced photocatalytic efficiency compared to unmodified ZnO. The current study focuses on the production of ZnO photocatalysts in conjunction with various composites. The synthesis of zinc oxide (ZnO) is first carried out using a chemical precipitation method characterized by its simplicity. Subsequently, ZnO is transformed using a photoreduction approach [43, 44].

The current study is focused on the hydrothermal synthesis of composite materials consisting of zingiber extract and ZnO. This composite material demonstrates an enhancement in photodegradation efficiency, particularly for the removal of antibiotics ofloxacin and RR141. In this study, a comparison was conducted between the results obtained and previous research studies, as presented in Table 1. This particular method is considered more appropriate for mass production of catalysts. This study presents a mechanism for synthesizing photocatalysts with exceptional efficiency in the context of environmental remediation.

**Table 1:** Comparison of RR141 azo dye ofloxacin antibiotic degradation using various photocatalysts.

Catalyst	Conc(mg)	Catalyst Amount	Light	Lamp	Time (min)	Photo (%)	Ref.
Photodegradation RR141							
SDS-capped ZnO	10 mg	50 mg	Uv	125 W	240	95	[6]
SDS-capped ZnO	10 mg	50 mg	Visible	15 W	240	60	[6]
Zingiber/ZnO-H	10 mg	50 mg	Uv	125 W	240	97	This Work
Zingiber/ZnO-H	10 mg	50 mg	Solar Light	-	240	99	This Work
Photodegradation Ofloxacin							
BiOCl	10 mg	50 mg	Uv	15 W	240	92	[10]
Bi2Mo6	10 mg	50 mg	Uv	15 W	240	89	[11]
Co3O4/TiO2/GO	10 mg	25 mg	Uv	300 W	90	91	[45]
Zingiber/ZnO-H	10 mg	50 mg	Uv	125 W	240	97	This Work
Zingiber/ZnO-H	10 mg	50 mg	Solar Light	-	240	95	This Work

## 2. Material and methods

### 2.1 Materials

*Zingiber Montanum* roots has been collect from Science Park Khon Kaen University which were collected in March 2023, ethyl alcohol 95%, denatured has been order from Daejung chemichals & materials Co., LTD, South Kore, Zinc Oxide has been order from MiniScience Company LLC. USA, Hydrothermal autoclave that has been used with model KH-200, with working pressure < = 3Mpa and all chemical reagents in this research are analytical grade.

### 2.2 Methods

#### 2.2.1. Preparation of the Zingiber montanum root extract

The procedure for obtaining the *Zingiber montanum* root extract was carried out as follows. The acquisition of fresh ginger was made from the local market. The item undergoes a comprehensive cleansing process in order to eliminate any potential contaminants. The roots were desiccated through the elimination of all liquid. The exocarp of the desiccated ginger was removed. The specimen was divided into smaller fragments and thereafter placed on oven, where it was subjected to a temperature of 70°C for a duration of 24 hours. 100 gram of the roots that had undergone complete desiccation were subjected to crushing using a mortar and subsequently pulverized. The addition of 500 mL of deionized water was performed gradually, while the pounding process was continuing. Subsequently, the mixture was subjected to filtration using no.1 Whatman paper and subsequently stored at a temperature of 4°C.

#### 2.2.2. Synthesis of Zingiber/Zinc oxide by hydrothermal (Zingiber/ZnO-H)

A hydrothermal autoclave with the size 200 mL was utilized to combine 100 ml of *Zingiber Montanum* with 0.1 grams of Zinc Oxide. The synthesis process was conducted using a hydrothermal approach, wherein the sample was subjected to heating in an oven at a temperature of 180°C for a duration of 3 hours. Following the process of synthesis, it is recommended to subject the substance to a drying period of 24 hours within an oven, resulting in the transformation of the material into a powdered form.

#### 2.2.3. Synthesis Zingiber/ZnO by precipitation methods (Zingiber/ZnO-P)

A solution containing 100 milliliters of *Zingiber Montanum* was combined with 0.1 grams of Zinc Oxide. The synthesis procedure was conducted using stirrer methods at room temperature for a duration of 24 hours. Prior to this, the Zingiber/ZnO-P mixture was dried in an oven for 24 hours till it transformed into a powdered form.

#### 2.2.4 Synthesis Zingiber/ZnO-H400 and Zingiber /ZnO-P400

1 gram of each powder (Zingiber/ZnO-H and Zingiber/ZnO-P) has been prepared and heated in the 400°C for 4hour [46].

#### 2.2.5 Photodegradation of the pollutants RR141 azo dye and ofloxacin

The photoactivity of the catalyst was evaluated by measuring the degradation of the azo dye RR141 and the antibiotic Ofloxacin (OFL Antibiotics). After preparation of 200ml with concentration 10 ppm of each pollutant, 50 mg of catalyst powder has been added and mixed in the room temperature of faculty of science, department chemistry, khon kaen university, Thailand, the catalyst has been mixed by using magnetic stirrer, 100 rpm under UV light (mercury lamp, 125 W), the distance between the UV lamp and the beaker glass is 30 cm, after preparation a dark condition in the duration of 60 minutes has been applied in the absence of light for photo irradiation purposes, 5 mL samples were taken within a certain period of time and centrifuged at a speed of 10,000 revolutions per minute (RPM) for 5 minutes. A centrifugation procedure was carried out to obtain the supernatant, which was then analyzed for concentration using UV-vis spectrophotometry techniques, by measuring absorbance at wavelengths of 544 nm (RR141) and 293 nm (OFL Antibiotics), respectively. After obtaining the absorbance, the photodegradation efficiency evaluation involves using the photodegradation efficiency equation, which is expressed as photoactivity (%) = ((1—C/C0) x 100%). This equation calculates the degradation percentage by comparing the initial concentration (C0) of an aqueous solution of a pollutant with its concentration (C) after a certain duration of irradiation. Pollutant degradation via photocatalysis can be characterized by a kinetic model which can be expressed using the first order reaction rate equation, ln (C0/C) = k1t. In this equation, k1 is the first-order reaction rate constant. This study centers on analyzing the influence of many experimental factors, including the initial pH of the solution, the amount of photocatalyst used, and the initial concentration of the pollutant, on the photoactivity observed during the investigation of dye and antibiotic degradation. This study introduced individual scavengers, including isopropyl alcohol (IPA), EDTA-2Na, and K_2_Cr_2_O_7_, to explore the key species responsible for pollutant removal. These scavengers are used for the specific purpose of neutralizing hydroxyl radicals, superoxide anion radicals, and electrons. Different scavengers were introduced separately in the presence of pre-prepared photocatalysts [1, 10].

#### 2.2.6 Photodegradation of RR141 and ofloxacin antibiotics in the presence of sunlight

The photodegradation of the antibiotics RR141 and OFL under natural uv sunlight. The RR141 and OFL Antibiotics solution was exposed to sunlight on the rooftop of Science Building Number 04, which is part of the Faculty of Science at Khon Kaen University in Thailand, in 21 April 2023. (latitude 1628′ 33.7′′N and longitude 10249′ 26.2′′E.

## 3. Results and discussions

### 3.1 Characterization of Zingiber and various conditions Zingiber/ZnO

**Fig 1:**
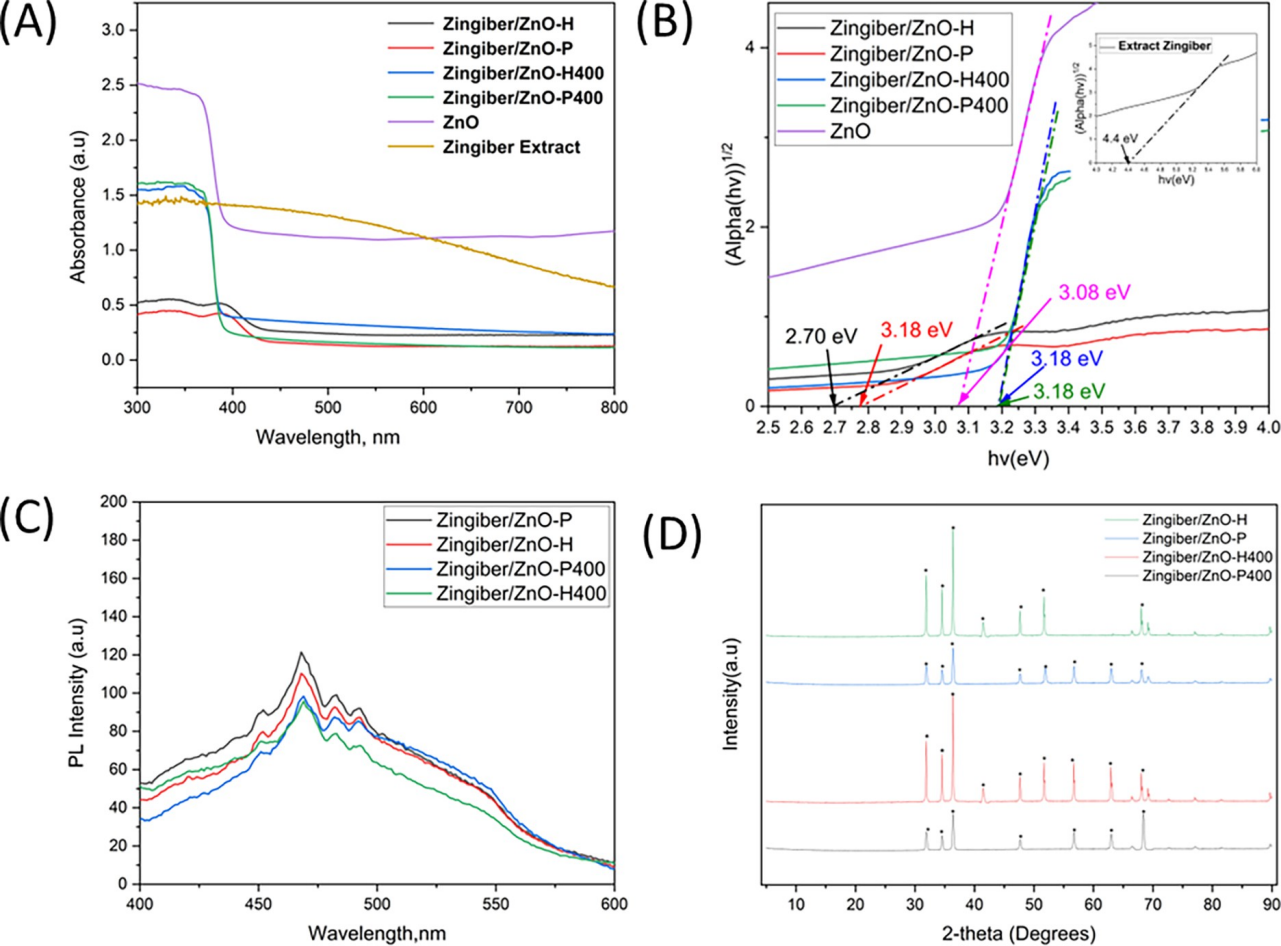
Characteristic of Catalyst Powder under various methods: (A) Diffused reflectance spectra (B) Tauc plot of band gap energy (C) Fluorescence spectroscopy spectra, and (D) X-Ray Diffraction analysis (XRD) of various condition of Zingiber/ZnO.

Fig 1A depicts the UV-vis diffuse reflectance spectra of the samples. The band gap energy of each sample was calculated using the Kubelka-Munk formula. This is depicted in Fig 1B. As a result, in the instance of ZnO, absorption edge values of 380 nm were recorded. The band gap values of bare Zinc Oxide, Zingiber Extract, Zingiber/ZnO-H, Zingiber/ZnO-P, Zingiber/ZnO-H400, and Zingiber/ZnO-P400, respectively, were determined to be 3.08, 4.4, 2.70, 2.77, 3.18, and 3.18 eV. The photoluminescence (PL) spectra shown in Fig 1C show that the Zingiber/ZnO-H400 photocatalyst had the lowest intensity in the PL spectra when compared to the other testing circumstances. This finding indicates that the Zingiber/ZnO-H400 photocatalyst has the lowest charge carrier recombination rate. The XRD data of Zingiber/ZnO in various synthesis conditions shows that Zingiber/ZnO-H, Zingiber/ZnO-P, Zingiber/ZnO-H400, and Zingiber/ZnO-P400 (Fig 1D) have a strong crystal peak and show diffraction peaks of ZnO at 2 = 31.4, 34.3, 36.6, 48.1, 56.6, 63.7, and The average crystallite size of Zingiber, Zingiber/ZnO-H, Zingiber/ZnO-P, Zingiber/ZnO-H400, and Zingiber/ZnO-P400 is 31.3 nm, 38.1 nm, 40.9 nm, and 36.9 nm, respectively, from XRD data. The particle size of these compounds directly influences the magnitude of the energy bandgap mentioned in Fig 1B. The addition of ZnO in conjunction with Zigiber extract exhibits a quantum confinement effect, wherein the dimensions of the material are directly proportionate to the wavelength of the electrons. The confinement of particles can have an impact on the arrangement of energy levels in the band structure, leading to a decrease in the band gap [46].

**Fig 2:**
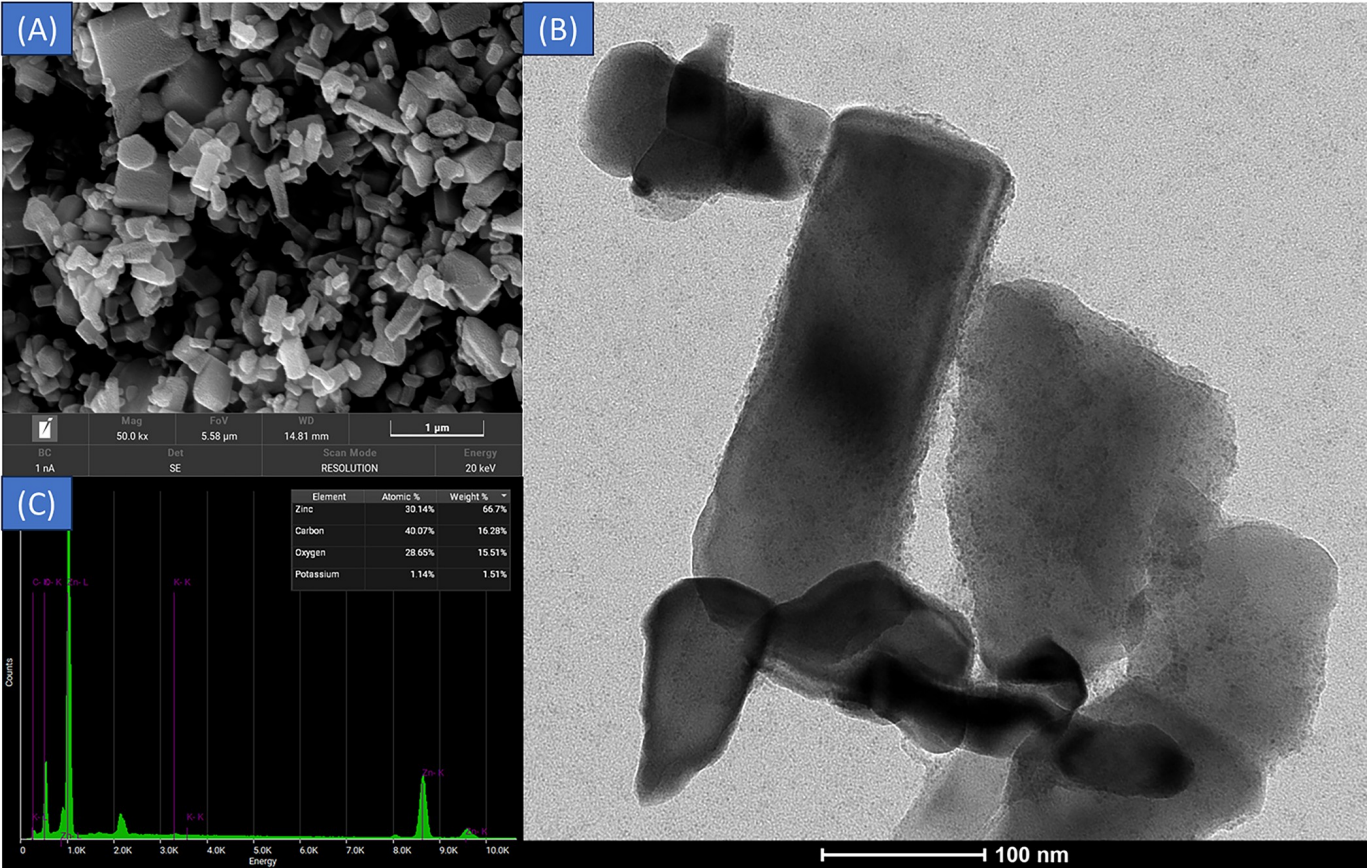
Characteristic of Catalyst Powder under various methods: (A) FE-SEM Data of Zingiber/ZnO-H, (B) TEM Data of Zingiber/ZnO-H, and (C) EDX of Zingiber/ZnO-H.

Transmission electron microscopy (TEM) and field emission scanning electron microscopy (FE-SEM) were used to examine the morphological structures of Zingiber and Zingiber/ZnO-H. Fig 2A shows the FE-SEM of Zingiber/ZnO-H after the extract of Zingiber was combined with zinc oxide in a hydrothermal setting. Fig 2B depicts the particle size of Zingiber/ZnO-H under TEM and reveals a much larger particle size than the crystallite size measured by XRD analysis, Fig 2A and 2B also demonstrate the binding of the Zingiber extract to Zinc Oxide, due to the presence of functional groups such as carboxyl, amino, or hydroxyl groups. These groups can engage with the surface of zinc oxide via electrostatic interactions. Carboxyl groups present in the zingiber extract can create chemical interactions with zinc oxide surfaces by means of electrostatic attractions between the negatively charged carboxylate groups and positively charged zinc ions on the ZnO surface. This rise in size can be due to the aggregation of multiple Zingiber/ZnO-H crystallites. Furthermore, the Zingiber/ZnO-H400 combination was analyzed for elemental structure using EDX. The spectra of Zingiber/ZnO-H400 (Fig 2C), revealed that Zingiber contains elements of Zinc (Zn), Carbon (C), and Oxygen (O), with atomic percentages of 66.7%, 16.8%, and 15.51%, with weigth% 30.14%, 40.07%, and 28.56%, respectively. The element stoichiometry Zingiber/ZnO-H400 result revealed a 3.9:1 atomic ratio of Zn to C and a 4.3:1 atomic ratio of Zn to O.

**Fig 3:**
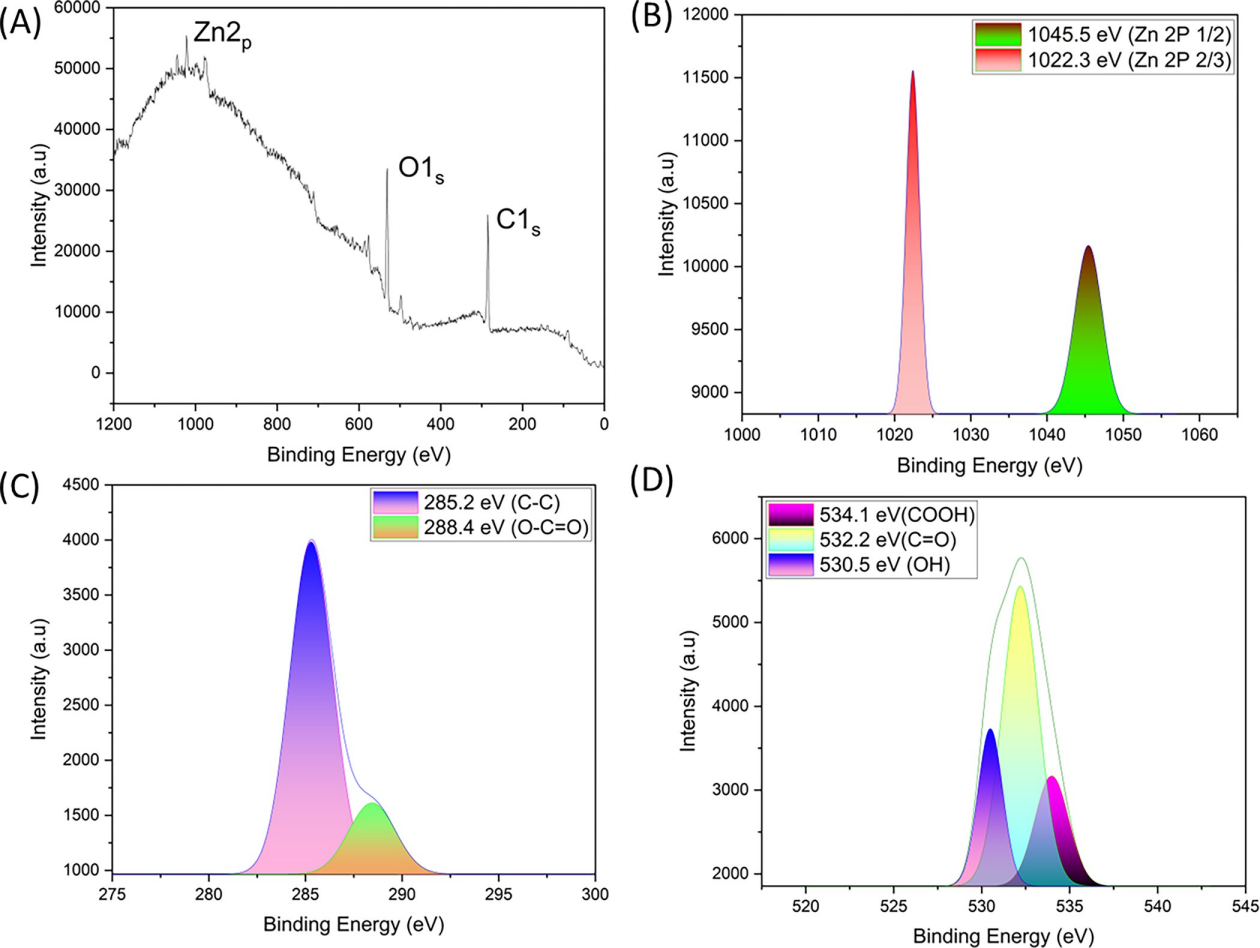
Characteristic of Catalyst Powder Zingiber/ZnO-H by XPS: (A) Survey Spectrum (B) Zn 2P peak (C) C1s peak and D (D) O1s peak.

X-ray photoelectron spectroscopy (XPS) was used to determine the elemental composition and chemical oxidation state of Zingiber/ZnO-H at the surface. Fig 3 depicts the interconnected spectra. Fig 3A shows the presence of Zn 2p, O1s, and C1s elements in the Zingiber/ZnO-H sample survey scan spectra. Deconvolution was conducted on the spectra illustrated in Fig 3B, resulting in the discovery of two separate peaks emanating from the clean ZnO material. These peaks were discovered to be located at 1022.3 eV for Zn 2p2/3 states and 1045.5 eV for Zn 2p 1/2 states, respectively. Fig 3C depicts the resolution of the C 1s spectra after deconvolution, which resulted in the detection of two distinct peaks at 285.2 eV and 288.4 eV. These peaks are caused by the presence of C-C and O-C = O bonds, respectively. Fig 3D shows the O 1s spectrum with a high level of resolution. Deconvolution analysis revealed three unique peaks situated at 534.1 eV (COOH), 532.2 eV (C = O), and 530.5 eV (OH) [10, 35, 47–49].

### 3.2 Photoactivity

The photocatalytic efficacy of each photocatalyst powder was assessed by examining the photodegradation of the RR141 azo dye and the antibiotic ofloxacin. The RR141 and OFL antibiotic concentrations were determined by using their respective max values of 544 nm and 293 nm, respectively. In addition, the photodegradation of OFL Antibiotics and RR141 in the presence of sunshine was investigated.

#### 3.2.1 Photodegradation of RR141 and OFL antibiotics under Uv-light

**Fig 4:**
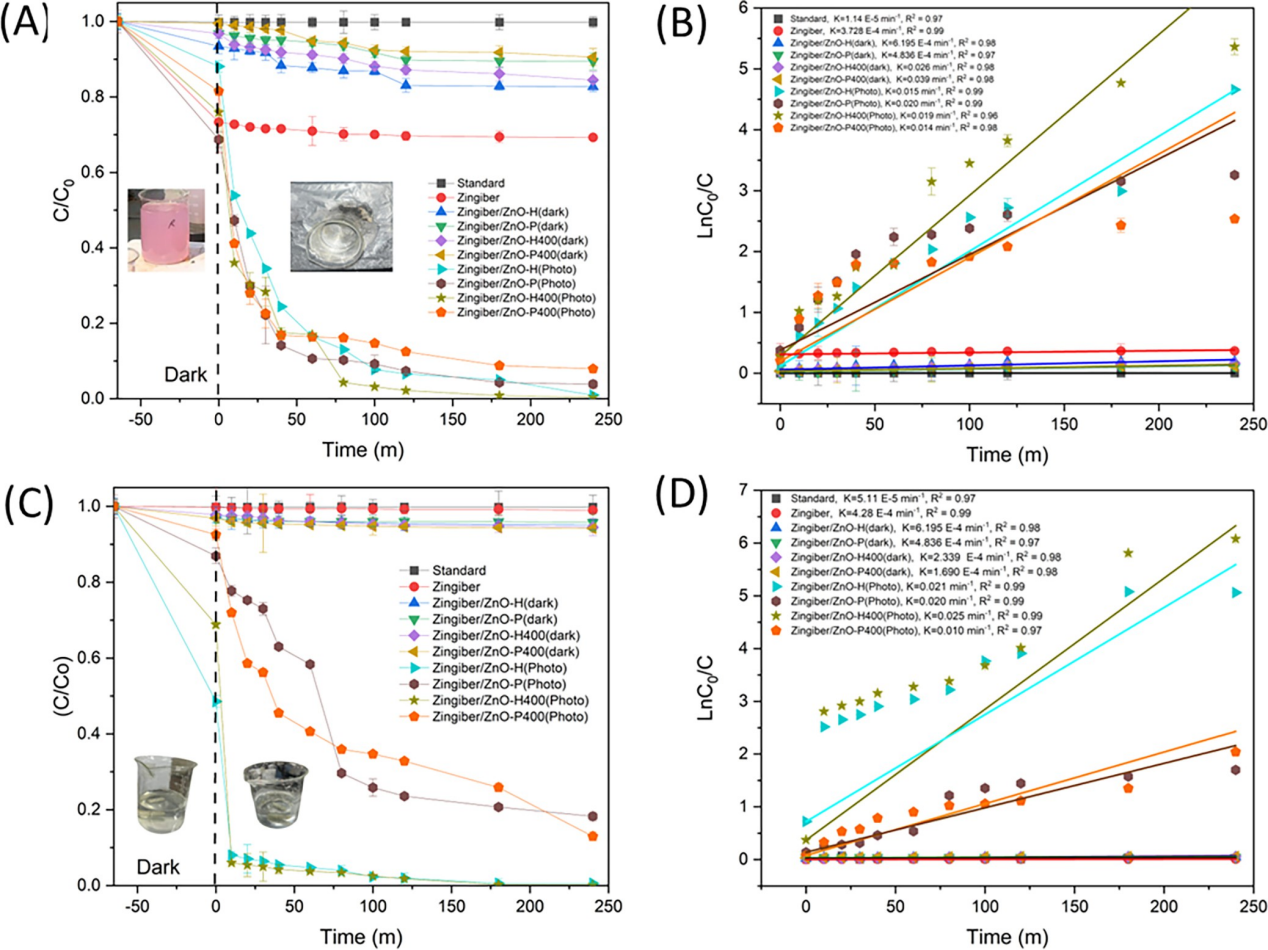
Photodegradation Comparison by used Uv Light in the various condition of CDs/ZnO: (A) RR141 C/C_0_ (B) RR141 LnC_0_/C (C) Ofloxacin C/C_0_ and (D) Ofloxacin LnC_0_/C, (SD, n = 3).

The analysis encompasses the investigation of the photodegradation process of RR141 dye, with particular emphasis on the temporal decline in dye concentration (C/C0) subsequent to UV light exposure, as depicted in Fig 4A. The adsorption technique demonstrated a prompt and efficient removal rate of the RR141 dye. The inclusion of Zingiber/ZnO-H, Zingiber/ZnO-P, Zingiber/ZnO-H400, and Zingiber/ZnO-P400 led to a gradual reduction in C/C0 over a period of time when exposed to UV irradiation, as depicted in Fig 4A. The utilisation of Zingiber/ZnO-H led to a notable reduction in C/C0, decreasing from 1 to 0.01 during a duration of 240 minutes. This observation suggests a degrading efficiency of 95% for RR141 when employing Zingiber/ZnO-H. The nanocomposite demonstrated a notable photodegradation efficiency of 95% with respect to the RR141 dye. The evaluation of the photoactivity of the catalysts was also performed by assessing the pace at which the photodegradation reaction occurred. The rate constants (k) for the photodegradation of Zingiber/ZnO-H, Zingiber/ZnO-P, Zingiber/ZnO-H400, and Zingiber/ZnO-P400 are determined to be 0.015, 0.020, 0.019, and 0.014 (Fig 4B), respectively. The corresponding coefficient of determination (R^2^) values for these degradation reactions are found to be 0.99, 0.99, 0.96, and 0.98. Upon determining the optimal conditions for the catalyst powder (Zingiber/ZnO-H). The objective of this study was to investigate the photodegradation of OFL antibiotics by examining the concentration of OFL antibiotics (C/C_0_) after exposure to UV light. Additionally, the research also focused on the removal of OFL antibiotics by an adsorption method. The C/C0 ratio exhibited a decline as the duration of UV irradiation increased for the integration of Zingiber/ZnO-H, Zingiber/ZnO-P, Zingiber/ZnO-H400, and Zingiber/ZnO-P400, as depicted in Fig 4C. The utilisation of Zingiber/ZnO-H resulted in a significant reduction in the C/C0 ratio, decreasing from 1 to 0.004 during a period of 240 minutes. The degradation efficiency of the OFL antibiotic is seen to be 99% when utilising Zingiber/ZnO-H. The nanocomposite facilitated a 99% photodegradation of ofloxacin. The Fig 4D illustrates the photodegradation rate constant (k) of Zingiber/ZnO-H. The photocatalyst exhibited a UV-driven photodegradation rate constant (k) of 0.02, with a coefficient of determination (R^2^) of 0.99.

#### 3.2.2 Photodegradation of RR141 and OFL antibiotics under sunlight

**Fig 5:**
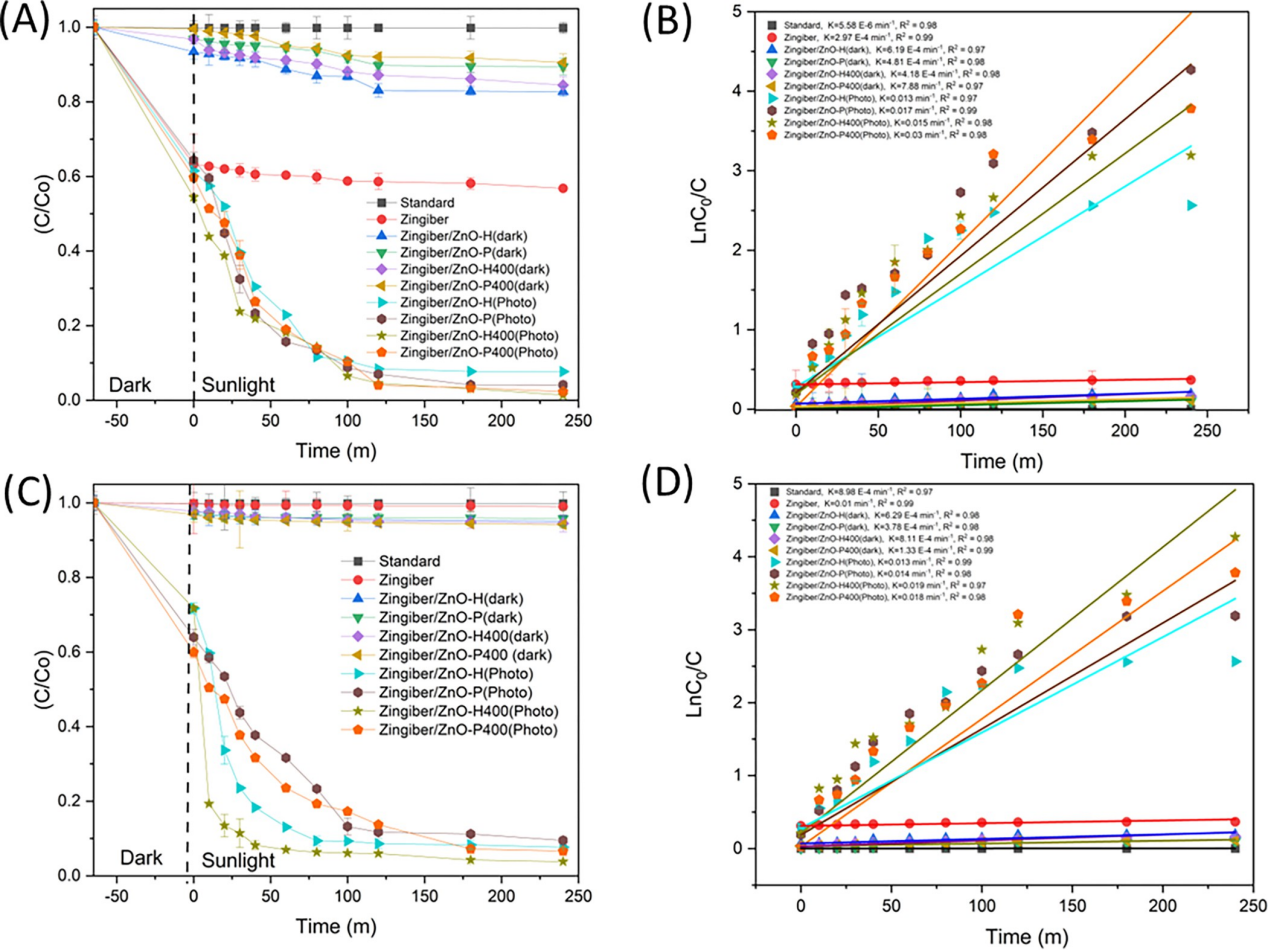
Photodegradation Comparison by used Sunlight in the various condition of CDs/ZnO: (A) RR141 C/C_0_ (B) RR141 LnC_0_/C (C)Ofloxacin C/C_0_ and (D) Ofloxacin LnC_0_/C, (SD, n = 3).

The investigation focused on assessing the photodegradation of RR141 through uv irradiation under sunshine, with the concentration (C/C0) of RR141 being the primary parameter of interest. Fig 5 illustrates the rapid elimination of the dye RR141 during this process. The C/C0 ratio exhibited a decline as the UV irradiation time increased for the inclusion of Zingiber/ZnO-H, Zingiber/ZnO-P, Zingiber/ZnO-H400, and Zingiber/ZnO-P400 (Fig 5A). After 240 minutes, the values reached 1 and 0.07, respectively. The study observed degradation efficiencies of 92%, 97%, 99%, and 97% for RR141 when employing Zingiber/ZnO-H, Zingiber/ZnO-P, Zingiber/ZnO-H400, and Zingiber/ZnO-P400, respectively. The catalysts’ photoactivity was evaluated using the quantification of the photodegradation reaction rate. The UV-induced photodegradation rate constants (k) for the Zingiber/ZnO-H, Zingiber/ZnO-P, Zingiber/ZnO-H400, and Zingiber/ZnO-P400 photocatalysts were determined to be 0.013, 0.017, 0.015, and 0.03, respectively, as depicted in Fig 5B.

**Fig 6:**
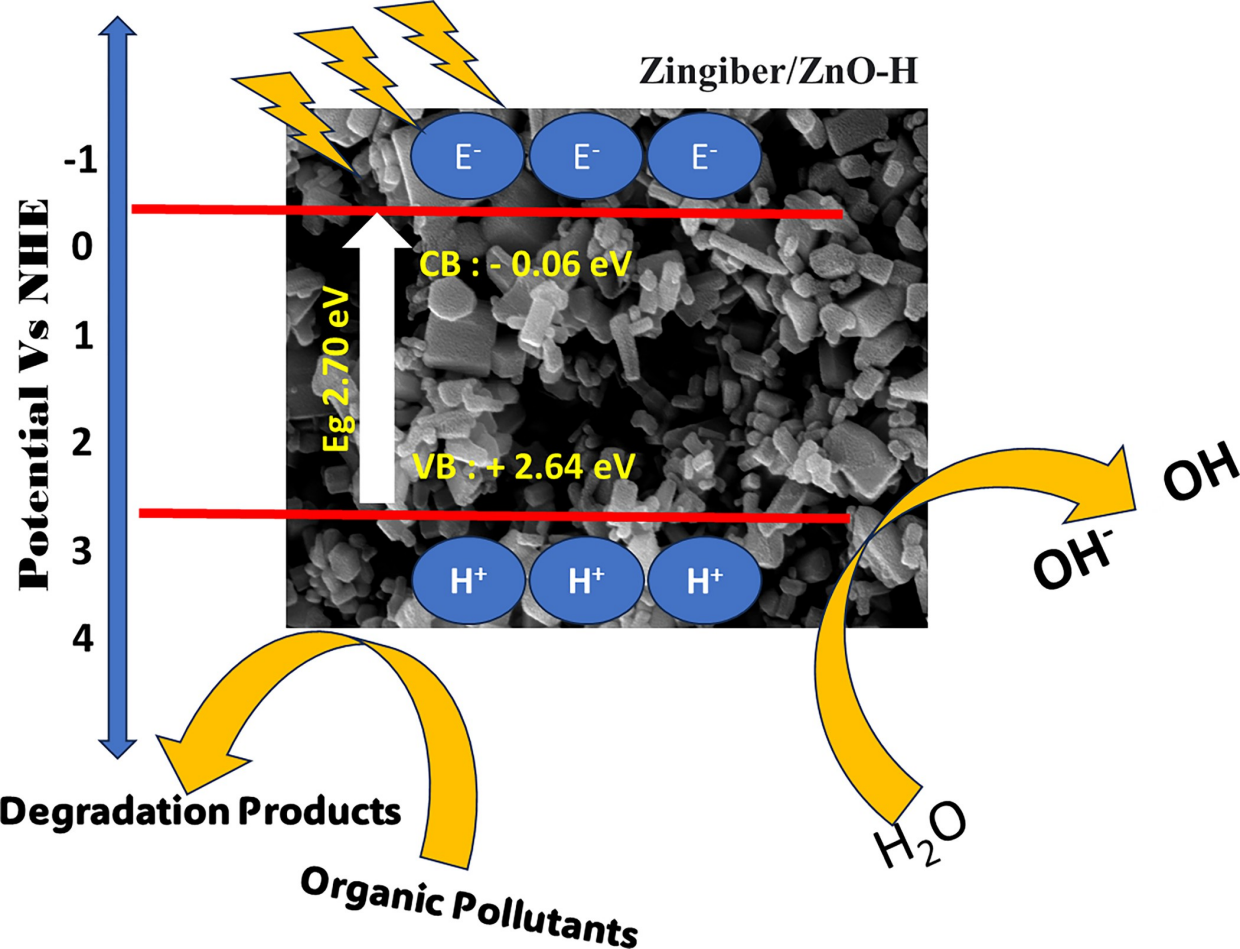
Schematic illustrations of the energy band structures of Zingiber/ZnO-H.

The study aimed to investigate the impact of ultraviolet (UV) irradiation from sunshine on the photodegradation of OFL Antibiotics, as depicted in Fig 5C. Furthermore, the elimination of the OFL Antibiotics exhibits a moderate effect following the introduction of Zingiber/ZnO-H, Zingiber/ZnO-P, Zingiber/ZnO-H400, and Zingiber/ZnO-P400. This is evidenced by a decrease in the ratio of C/C0 with increasing UV irradiation time (as shown in Fig 5C), with values decreasing from 1 to 0.07, 0.09, 0.03, and 0.06 correspondingly, after a duration of 240 minutes. The degradation efficiency of the antibiotic OFL Antibiotics was assessed using several catalysts, namely Zingiber/ZnO-H, Zingiber/ZnO-P, Zingiber/ZnO-H400, and Zingiber/ZnO-P400. The results indicated degradation efficiencies of 92%, 90%, 96%, and 93% respectively. These findings suggest that the optimal condition for degradation is achieved when utilising Zingiber/ZnO-H400. The assessment of the photoactivity of the catalysts was also carried out by evaluating the rate of the photodegradation reaction. In Fig 5D, the photodegradation rate constants (k) for Zingiber/ZnO-H, Zingiber/ZnO-P, Zingiber/ZnO-H400, and Zingiber/ZnO-P400 were seen to be 0.013, 0.014, 0.019, and 0.018, respectively. The corresponding R^2^ values for these samples were found to be 0.99, 0.96, 0.97, and 0.98. photodegradation mechanism of Zingiber/ZnO-H heterojunction. explained by The phenomenon of electron and hole creation in the conduction band (C_B_) and valence band (V_B_), respectively, was found when subjected to light irradiation. Consequently, the production of reactive species is expected. The energy levels of the conduction band (C_B_) and valence band (V_B_) edges in Carbon Dots (Zingiber) and zinc oxide (ZnO) (Zingiber/ZnO-H) photocatalysts may be determined using the Mulliken electronegativity theory. This theory states that the energy of the valence band edge (E_VB_) can be calculated as the difference between the electronegativity of the material (χ), the energy of the conduction band (E_C_), and half of the band gap energy (E_g_). Similarly, the energy of the conduction band edge (E_CB_) can be obtained by subtracting the band gap energy (Eg) from the energy of the valence band edge (E_VB_). The valence band potential is denoted as E_VB_, while the conduction band potential is represented by E_CB_. The normal hydrogen electrode potential, which is around 4.5 eV, is symbolised as E_C_. Additionally, the absolute value of the electronegativity of the semi-conducting photocatalyst is denoted as χ. The electronegativity values of zinc oxide (ZnO) were determined to be 5.79 electron volts (eV) per atom. Based on the theoretical framework (E_vb_ = χ –E_c_ +0.5 E_g_ and E_cb_ = E_vb—_E_g_) the determination of the energy valence band (E_VB_) and energy conduction band (E_CB_) potentials of ZnO yielded values of 2.64 and -0.06 eV, respectively (Fig 6). The process of creating heterostructures by coupling two photocatalysts has been proven as a means to determine heterojunction photocatalysts. Previous proposals have been made for Type II heterojunctions and Z schemes. Nevertheless, it is acknowledged that both conventional Z-scheme photocatalysts and solid-state Z-scheme photocatalysts still exhibit some deficiencies in their electron transport processes. In this instance, a package with a high redox capacity is achieved. Collectively, one achieves seamless photoabsorption, exceptional delivery, and a robust redox potential in principle. The use of this heterojunction process is quite effective in this research. The process of photocatalytic destruction of the harmful pollutant may be described,

$$Zingiber/ZnO+hv\to Zingiber/ZnO+e^{-}+h^{+}$$ (1)

$$e^{-}+O2\to •{O_{2}}^{-}$$ (2)

$$•{O_{2}}^{-}+2H_{2}O+e^{-}\to 2^{•}OH+2OH^{-}$$ (3)

$$h^{+}+OH^{-}\to •OH$$ (4)

•OH+ContaminantPollutant→products (5)

$$h^{+}+Contaminant\;Pollutant\to products$$ (6)

#### 3.2.3 The impact of experimental parameters on photoactivity

**Fig 7:**
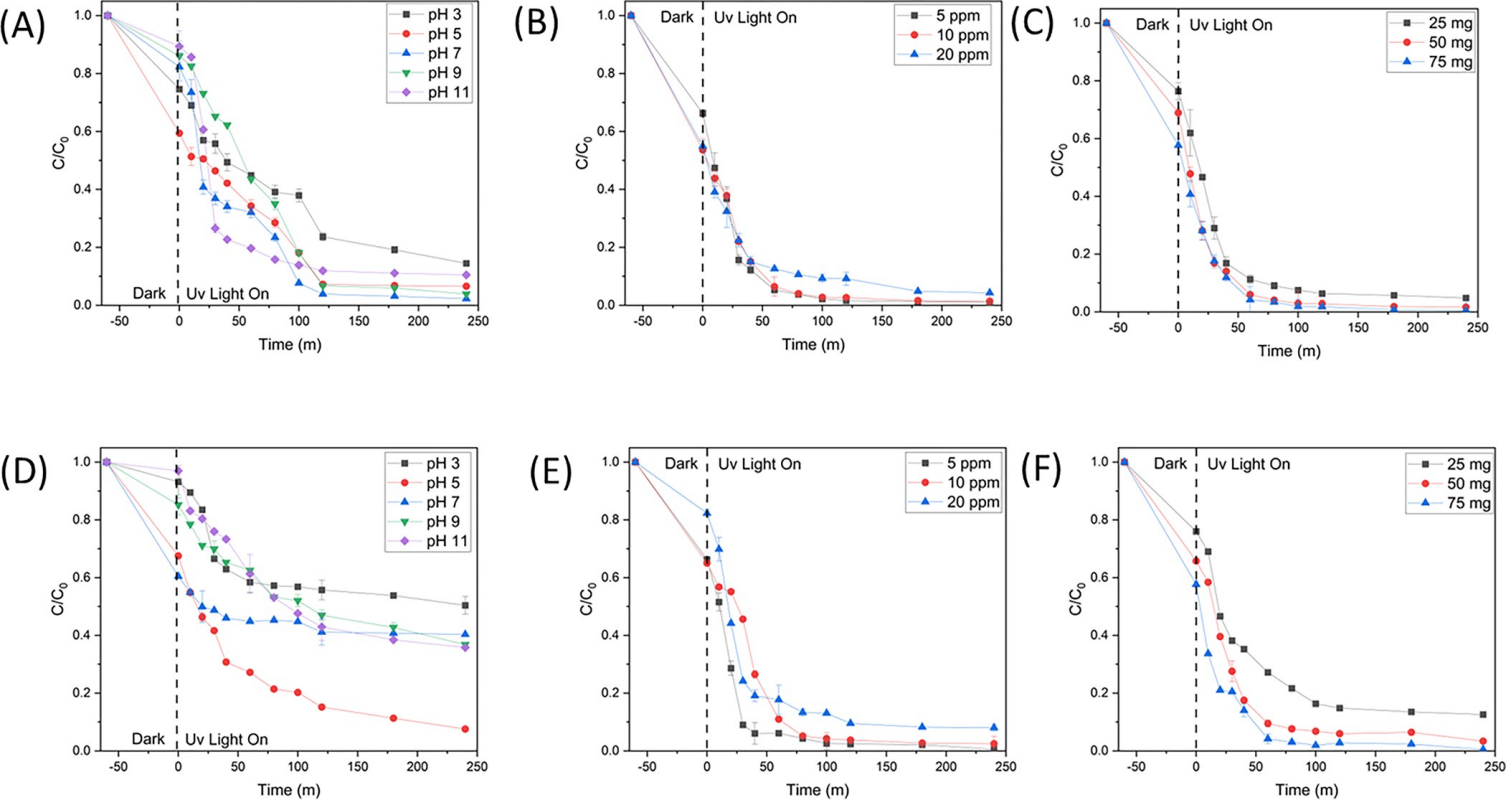
Various condition test to get the optimum condition: (A)The effect of various conditions of pH (B) catalyst concentration and (C) pollutant concentration for degradation of RR141, (D) Various conditions of pH, (E) catalyst concentration and (F) pollutant concentration for degradation of Ofloxacin. (SD, n = 3).

The experiment was conducted to determine the optimal circumstances for conducting the test. These conditions included investigating the impact of pH, catalyst concentration, and RR141 concentration (Fig 7). According to Fig 7A, the inclusion of a pH7 buffer exhibits superior performance compared to the other conditions, yielding an efficiency of 98%. In the present study, it was observed that the optimal catalyst concentration was determined to be 75 mg, as depicted in Fig 7B. Additionally, the optimal concentration of RR141 was found to be 5 ppmx, as illustrated in Fig 7C. Fig 7D demonstrates the examination of the influence of the experimental variables on the degradation of OFL antibiotics. The pH of the OFL antibiotic solution is around 7. On the contrary, within the pH range of 9 to 11, the repulsion between the anionic OFL antibiotic and the negatively charged surface of the photocatalyst occurs. A potential consequence of a reduction in the adsorption capacity of OFL antibiotics onto the surface of the photocatalyst is a corresponding drop in the catalytic efficacy of the photocatalyst. The increased adsorption of anionic OFL on the positively charged surface of the photocatalyst can be attributed to the higher photoactivity seen within the pH range of 5.5–6. It is important to acknowledge that the photocatalyst has the potential to disintegrate under highly acidic circumstances, typically characterized by a pH of approximately 3. The impact of the photocatalyst content on the photodegradation of OFL was also examined (Fig 7E). The augmentation of photoactivity is attributed to the rise in the quantity of OFL molecules adsorbed on the surface of the photocatalyst and the increase in the density of photocatalyst particles per unit area of photo irradiation, resulting from the higher content of the photocatalyst. Nevertheless, the loss in photoactivity at a photocatalyst level of 75 mg can be attributed to the concurrent increase in solution turbidity, resulting in a reduction in photo penetration. The outcome will lead to a reduction in photoactivity. Furthermore, the impact of the concentration of OFL on photoactivity was determined (see Fig 7F). The introduction of OFL concentration has been seen to have a detrimental effect on the performance of the photocatalytic system. The absorption of light by OFL molecules exhibits a positive correlation with the concentration of OFL. This phenomenon may lead to a decrease in the incident light intensity on the surface of the photocatalyst, thereby compromising its overall efficiency by a factor of 8. With a concentration of 5 parts per million (ppm) of OFL. The photoactivities of the nanocomposite photocatalyst in the degradation of the antibiotic OFL under sunlight were also investigated in a previous study.

#### 3.2.3 The effect of trapping agent and reusability

**Fig 8:**
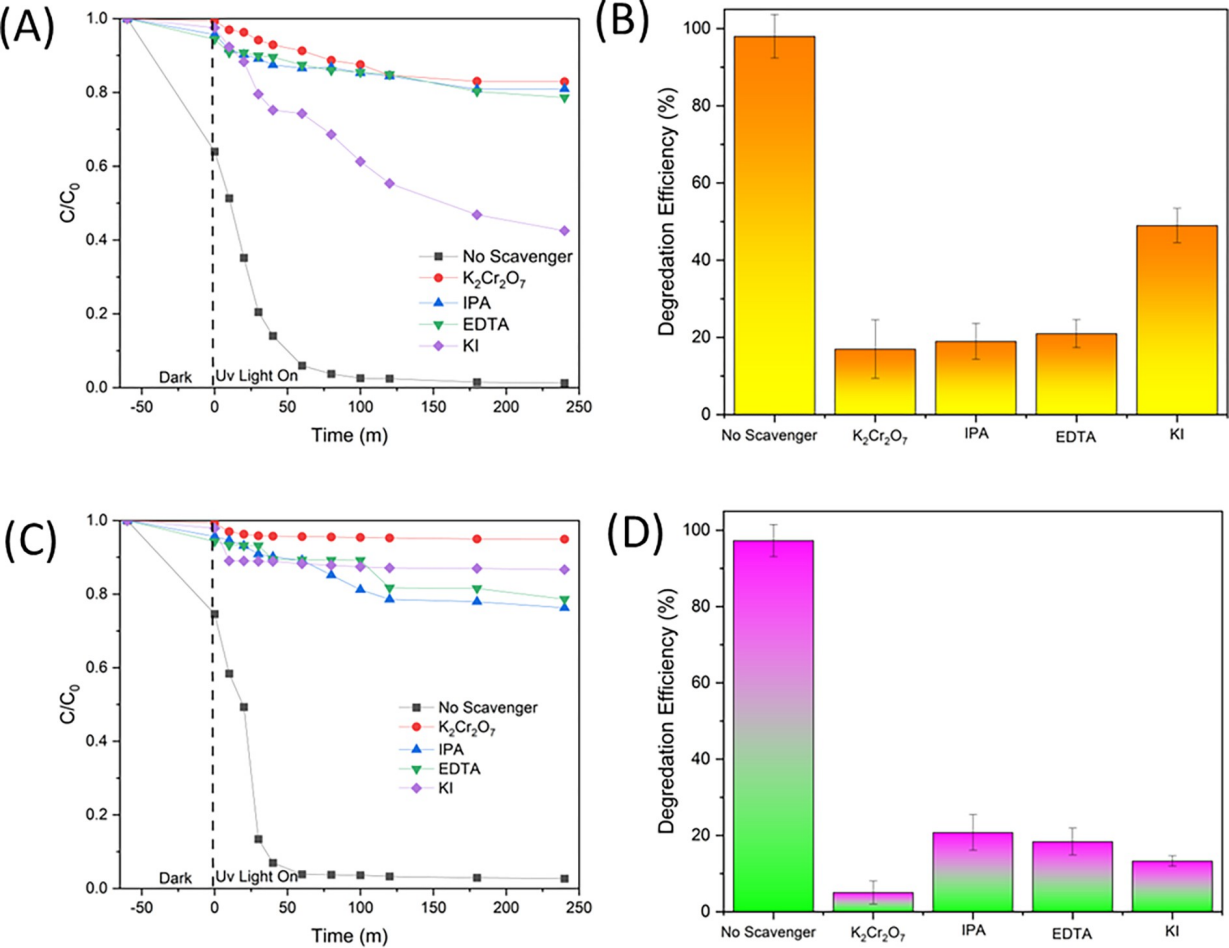
Effect of various scavenger in the degradation of pollutant: (A) The Concentration (C0/C) by photodegradation on ofloxacin (B) degradation efficiency on ofloxacin (C) The Concentration (C0/C) by photodegradation on RR141 (D) degradation efficiency on RR141.

**Fig 9:**
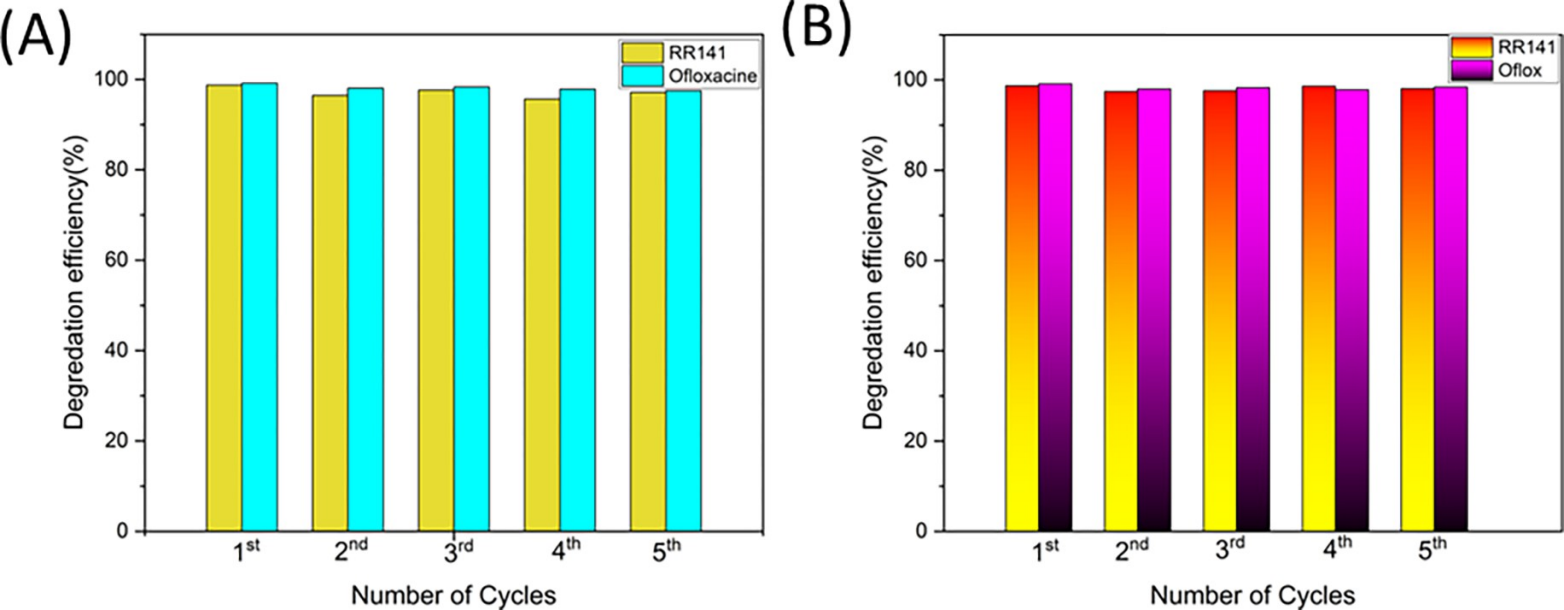
Repetition of photocatalyst toward degradation of RR141 dye and OFL antibiotic by using CDs/ZnO-H400: (A) Uv Light (B) Sun Light.

**Fig 10:**
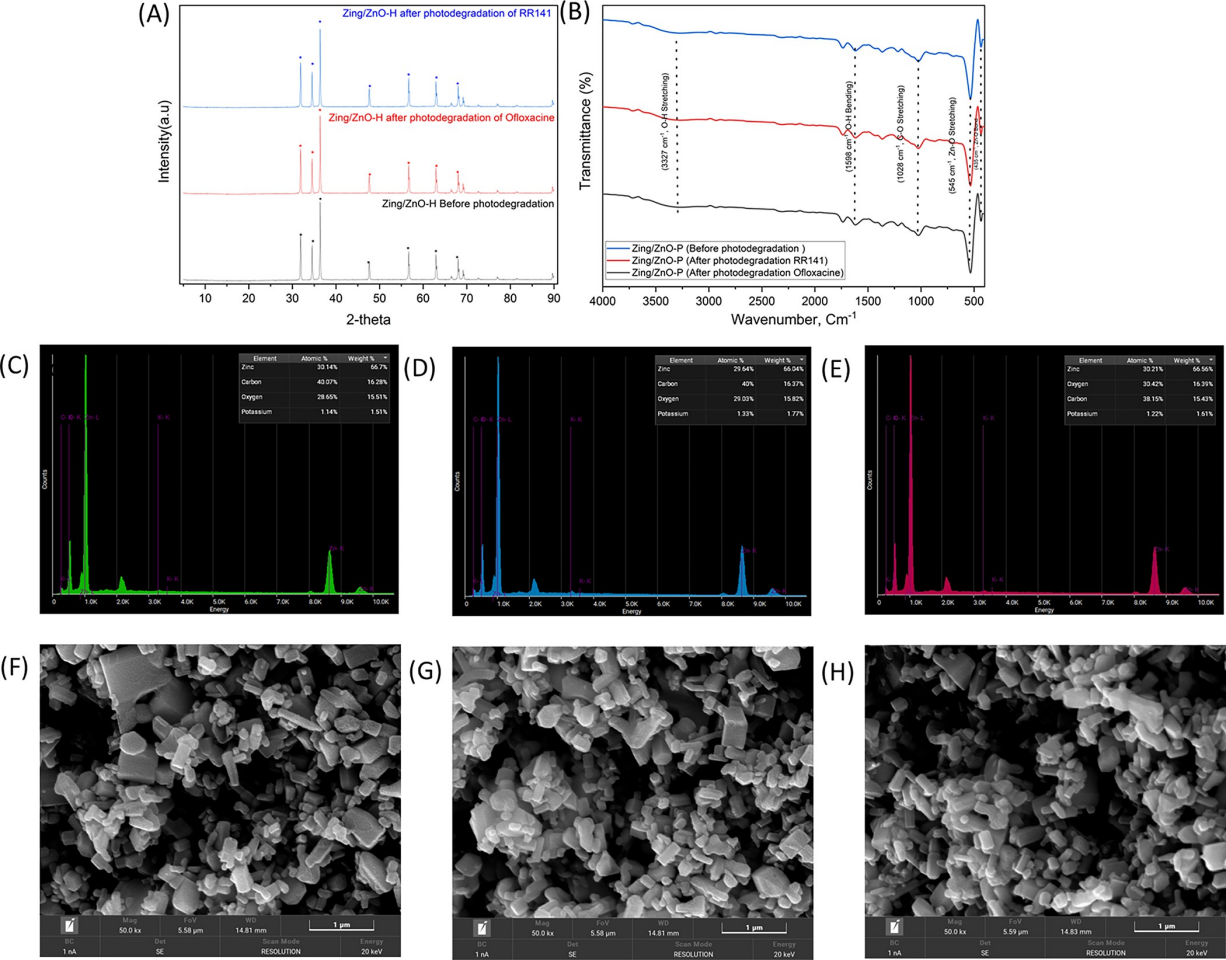
The effect before-after of catalyst powder under various methods: (A) XRD of Zingiber/ZnO-H and (B) FTIR of Zingiber/ZnO-H, EDX of (C) Zingiber/ZnO-H before photocat (D) Zingiber/ZnO-H after photocat RR141 (E) Zingiber/ZnO-H after photocat Oflox, and SEM of (F) Zingiber/ZnO-H before photocat (G) Zingiber/ZnO-H after photocat RR141 (H) Zingiber/ZnO-H after photocat Oflox.

The investigation of trapping agents in a trapping experiment has been conducted to examine the effectiveness of RR141 and OFL Antibiotics. This study examines the influence of specific reactive species on the degradation mechanism of the OFL antibiotic. In this study, the efficacy of various scavengers, namely EDTA, IPA, K_2_Cr_2_O_7_, and KI, was investigated in their role as quenchers of •OH, h+, and e–, respectively. Fig 8 demonstrates a notable decrease in photoactivity following the photochemical reaction involving potassium iodide (KI). The observed efficiency of the reaction was determined to be 47%, which was accompanied by a significantly low rate constant in comparison to the scenario where a scavenger mechanism was absent (Fig 8A and 8B). The photogenerated hole plays a pivotal role in the photodegradation process of OFL Antibiotics. An OFL antibiotic, similar to other OFL antibiotics, demonstrates comparable traits and properties. The experiment designated as RR141 aimed to assess the effectiveness of several scavengers. A notable drop in photoactivity was seen subsequent with IPA (Fig 8C and 8D). The efficacy of the scavenger process was found to be 19%, exhibiting a much lower rate constant in comparison to the absence of any scavenger process. The evaluation also encompassed an examination of the cycling capacity of the catalyst powder. The investigation into the elimination of the OFL antibiotic and RR14 dye was carried out through a series of five consecutive trials. After the initial phase of photocatalytic experimentation, the catalyst was filter by using whatman membrane filter no 1, and wash with Di water 3 times and dry in the oven 70°C before being applied in next cycles, up to the fifth cycle. The extraordinary cycling ability of the photocatalyst Zingiber/ZnO-H is demonstrated in the fifth run, as depicted in Fig 9. The photocatalyst’s ability to degrade RR141 exhibits a marginal reduction in efficacy, with a decline from 95% (4th run) to 99% (1st run) (Fig 9A). In a similar vein, the photocatalyst demonstrates a marginal decline in the degrading efficiency of OFL Antibiotics, with a fall from 97% in the fifth run to 99% in the first run (Fig 9B). The nanocomposite photocatalyst demonstrated a notable degree of cycling performance, thereby substantiating its commendable cycling capability. The degradation performance of RR141 and ofloxacin using Zingiber/ZnO-H cycling has also been investigated in the presence of sunshine. The photodegradation of RR141 by the photocatalyst demonstrates a slight reduction in efficiency, with a fall from 94% (3rd run) to 99% (1st run). Likewise, the photocatalyst demonstrates a marginal decline in the degrading efficiency of OFL Antibiotics, with a fall from 96% in the fourth run to 99% in the first run. The nanocomposite photocatalyst demonstrated a notable degree of cycling performance, thereby substantiating its commendable cycling capability. Additionally, Fig 10 provides empirical support for the structural integrity of the synthesised catalyst. The X-ray diffraction (XRD) analysis of Zingiber/ZnO-H, both before and after photochat, as shown in Fig 10A, reveals that the structural composition of the samples remains consistent. This suggests that the morphology of both the fresh and utilised Zingiber/ZnO-H samples is similar, indicating their stability. This observation is additionally substantiated by the resemblance in the X-ray diffraction (XRD) spectra prior to and subsequent to the photocatalytic process. Furthermore, the Fourier-transform infrared (FTIR) analysis (Fig 10B) and the energy-dispersive X-ray spectroscopy (EDX) analysis (Fig 10C–10E) demonstrate a comparable distribution of Zinc, Carbon, and Oxygen weights, respectively. The FE-SEM study, depicted in Fig 10F–10H, demonstrates a resemblance between the SEM images acquired prior to and following the photodegradation examination.

## 4. Conclusion

The catalyst powder is synthesized using hydrothermal techniques, wherein Zingiber montanum is combined with Zinc Oxide (ZnO). The findings were demonstrated in the present investigation. The Zingiber/ZnO-H photocatalyst exhibited promising catalytic activity in the reduction of RR141 and OFL antibiotics. This study also examined the impact of sunlight on degradation, revealing degradation rates of 97% for RR141 and 99% for ofloxacin under UV Lamp exposure, and degradation rates of 97% for RR141 and 95% for ofloxacin under Solar Light exposure. The primary facilitator of the breakdown of the harmful contaminant is hydroxyl radicals. The produced photocatalyst demonstrates remarkable stability and sustains its superior performance even after conducting five cycles, suggesting a promising potential for cycling capability. The findings of the present investigation suggest that the Zingiber/ZnO-H photocatalyst has promise in the realm of environmental preservation.
